# Supplementary material for: Temporal and spatial patterns of small vertebrate roadkill in a supercity of eastern China
Source: PeerJ. 2023 Oct 9;11:e16251. doi: 10.7717/peerj.16251 (PMC10569179; doi:10.7717/peerj.16251)
Supplement: Supplemental Information 1 — Clark-Evans index = Average observed Net-NND/Average expected Net-NND) (value > 1 for aggregated distribution, = 1 for random distribution, <1 for dispersed distribution [file peerj-11-16251-s001.docx]

Table A1. Results of the spatial distribution patterns analysis with the Clark-Evans index (Clark-Evans index = Average observed Net-NND/ Average expected Net-NND) (value > 1 for aggregated distribution, = 1 for random distribution, < 1 for dispersed distribution).

| **Road** | **Average observed Net-NND** | **Lower critical value for one-sided significance level** | **Upper critical value for one-sided significance level** | **Average expected Net-NND** | **Clark-Evans index** |
| --- | --- | --- | --- | --- | --- |
| G1 | 201.8121 | 187.1269 | 287.3964 | 239.0781 | 0.8441 |
| G2 | 662.0474 | 343.5598 | 864.1304 | 608.4068 | 1.0882 |
| G3 | 454.0626 | 400.6966 | 562.614 | 478.8284 | 0.9483 |
| S1 | 414.141 | 339.498 | 489.971 | 415.7036 | 0.9962 |
| S2 | 179.25 | 199.0228 | 332.3176 | 265.9827 | 0.6739 |
| S3 | 351.0704 | 353.2616 | 656.6516 | 507.7328 | 0.6914 |
| X1 | 194.1638 | 143.3366 | 243.0891 | 194.2998 | 0.9838 |
| X2 | 412.2508 | 256.6488 | 555.3932 | 407.5774 | 1.0115 |
| X3 | 609.07 | 452.7706 | 945.2231 | 696.936 | 0.8739 |
