# Supplementary figures and images for: Temporal and spatial patterns of small vertebrate roadkill in a supercity of eastern China
Source: PeerJ. 2023 Oct 9;11:e16251. doi: 10.7717/peerj.16251 (PMC10569179; doi:10.7717/peerj.16251)

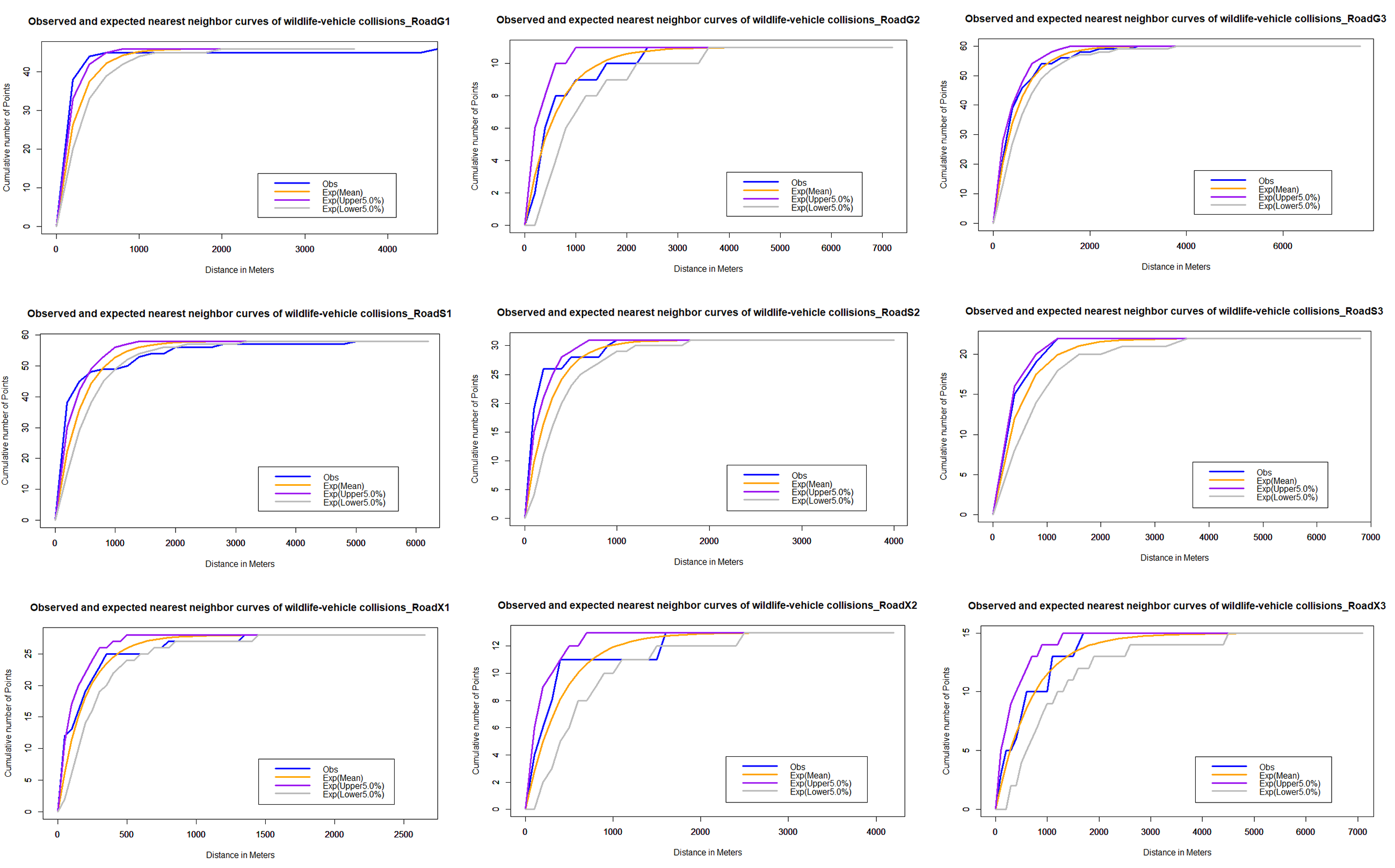

Supplement: Supplemental Information 3 — The blue line is the observed curve, the orange line is the expected curve, the purple line means the upper limit of the 95% confidence interval and the grey line means the lower limit of the 95% confidence interval. If the observed curve is above the upper confidence limit, the distribution pattern of roadkill can be considered to be clustered at the 95% confidence level; if it is below the lower confidence limit, the distribution pattern can be considered to be dispersed at the 95% confidence level; and if it is between the upper and lower curves, the assumption of complete spatial randomness (CSR) cannot be rejected at the 0.95 level of confidence. [file peerj-11-16251-s003.png]
